# Tumor lysis syndrome following letrozole for locally advanced breast cancer: a case report

**DOI:** 10.1186/s40792-024-01901-1

**Published:** 2024-04-24

**Authors:** Masayuki Kikuchi, Rika Miyabe, Hirokazu Matsushima, Hidenori Kita, Junko Kobayashi, Takashi Ando, Koji Atsuta, Tsunehiro Shintani

**Affiliations:** 1 Department of Surgery, Japanese Red Cross Shizuoka Hospital, 8-2, Otemachi, Aoi-ku, Shizuoka-shi, Shizuoka 420-0853 Japan; 2 Tosen Clinic, 1-20, Gohuku-cho, Aoi-ku, Shizuoka-shi, Shizuoka 420-0031 Japan

**Keywords:** Breast neoplasms, Letrozole, Tumor lysis syndrome

## Abstract

**Background:**

Letrozole, an aromatase inhibitor, is used to treat breast cancer in postmenopausal women. Tumor lysis syndrome (TLS) is a complication that can trigger multiple organ failure caused by the release of intracellular nucleic acids, phosphate, and potassium into the blood due to rapid tumor cell disintegration induced by drug therapy. TLS is uncommon in solid tumors and occurs primarily in patients receiving chemotherapy. Herein, we report a rare occurrence of TLS that developed in a patient with locally advanced breast cancer following treatment with letrozole.

**Case presentation:**

An 80-year-old woman with increased bleeding from a fist-sized left-sided breast mass presented to our hospital. Histological examination led to a diagnosis of invasive ductal carcinoma of the luminal type. The patient refused chemotherapy and was administered hormonal therapy with letrozole. Seven days after letrozole initiation, she complained of anorexia and diarrhea. Blood test results revealed elevated blood urea nitrogen (BUN) and creatinine (Cr) levels, and she was admitted to our hospital for intravenous infusions. On the second day after admission, marked elevations of LDH, BUN, Cr, potassium, calcium, and uric acid levels were observed. Furthermore, metabolic acidosis and prolonged coagulation capacity were observed. We suspected TLS and discontinued letrozole, and the patient was treated with hydration, febuxostat, and maintenance hemodialysis. On the third day after admission, her respiratory status worsened because of acute respiratory distress syndrome associated with hypercytokinemia, and she was intubated. On the fourth day after admission, her general condition did not improve, and she died.

**Conclusions:**

Although TLS typically occurs after chemotherapy initiation, the findings from the present case confirm that this syndrome can also occur after hormonal therapy initiation and should be treated with caution.

## Background

Letrozole, an aromatase inhibitor, is used to treat breast cancer in postmenopausal women. Tumor lysis syndrome (TLS) is a complication that can cause multiple organ failure due to the release of intracellular nucleic acids, phosphate, and potassium into the blood owing to rapid tumor cell disintegration triggered by drug therapy. TLS is most commonly reported in hematologic malignancies, but can also occur in solid tumors in rare cases. The frequency of TLS in solid tumors has been reported to be less than 0.3% [1]. In metastatic solid tumors, TLS occurs primarily in patients receiving chemotherapy, and only two TLS cases associated with letrozole have previously been reported [2, 3].

This case report aimed to document and analyze a rare occurrence of TLS that developed in a patient with locally advanced breast cancer following treatment with letrozole, a hormone therapy. This report delves into the clinical presentation, diagnostic processes, treatment interventions, and the unfortunate fatal outcome associated with TLS in this scenario.

## Case presentation

**Fig. 1:**
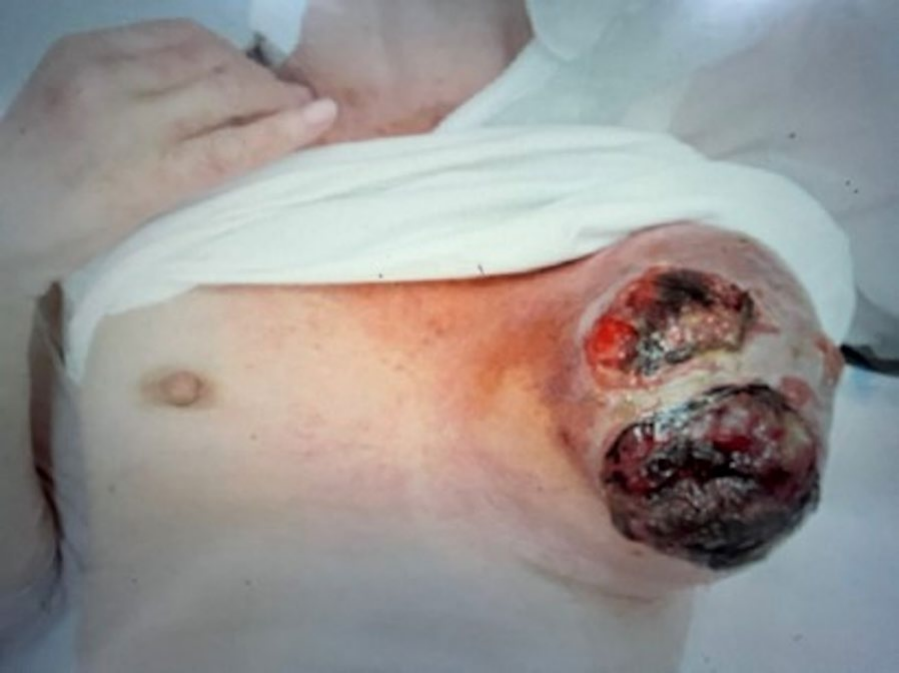
Patient photograph. The image shows the left breast lesion with irregular margins as well as necrotic and pyogenic lesions

An 80-year-old female patient with no prior medical history had been treated at another hospital for bleeding from a 10-cm-sized tumor in her left breast. Breast cancer was suspected, but she refused to undergo an examination. Six months later, the tumor grew even larger, and bleeding from the breast tumor did not stop; therefore, she visited our hospital. The left breast tumor was 12 × 10 cm in size, with hemorrhage and effusion noted on her clothing (Fig. 1). Hemostasis was achieved during the examination at our hospital. Blood tests revealed mild elevation of the blood urea nitrogen (BUN)/creatinine (Cr) ratio but no anemia, and tumor markers were normal (BUN, 30.7 mg/dl; Cr, 0.94 mg/dl, LDH, 134 U/L, carcinoembryonic antigen, 3.62 ng/ml; cancer antigen 15–3, 14 U/ml).

**Fig. 2:**
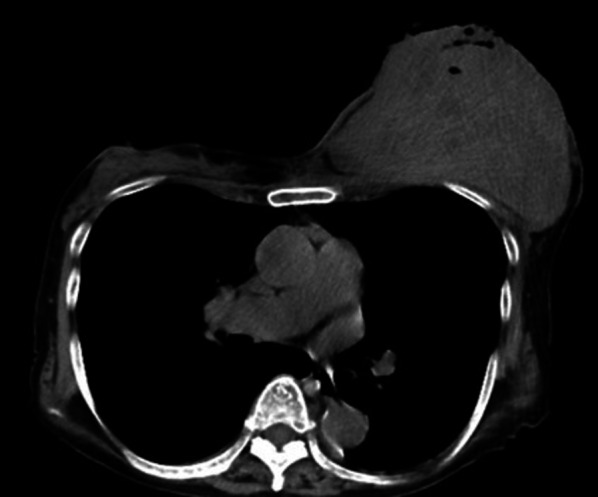
Breast computed tomography image. A 12 × 10-cm-sized mass was found in the left breast. No obvious pectoralis major muscle invasion was observed. Additionally, there was no significant lymph node swelling or findings suspicious of distant metastasis

Computed tomography revealed a 12 × 10 cm left breast mass, which was suspected to have invaded the epidermis and pectoralis major muscle. Axillary lymph nodes were not enlarged, and there was no obvious distant metastasis (Fig. 2).

Needle biopsy revealed a diagnosis of invasive ductal breast carcinoma (cT4bN0M0 cStageIIIB, estrogen receptor 50%, progesterone receptor 70%, human epidermal growth factor receptor 2 [HER2] 1 + , Ki67 26.16%), but most of the tissue was necrotic. The patient refused surgery or chemotherapy and was initiated on letrozole 2.5 mg/day. The bleeding area from the tumor was followed up with Mohs paste [4] and Rozex gel® (Mohs paste is not covered by insurance in Japan).

One week later, the patient complained of nausea and diarrhea. Blood tests revealed marked renal dysfunction and hyperuricemia, and she was admitted to the hospital for intravenous infusions. On the second day after admission, blood tests revealed further renal function deterioration, marked metabolic acidosis, and prolonged coagulopathy (Table 1). There were no arrhythmias or clinical findings suggestive of seizures or pulmonary emboli. The left breast tumor had shrunk and the surface tumor had self-destructed due to necrosis. Therefore, TLS and associated disseminated intravascular coagulation syndrome associated with tumor shrinkage with letrozole were suspected. High-volume rehydration, rasburicase for the hyperuricemia, and hemodialysis were initiated. On the third day after admission, the acidosis did not improve, and the patient developed respiratory distress and impaired consciousness. The patient refused intubation and underwent bilevel positive airway pressure ventilation. Although hemodialysis was performed daily, the patient’s general condition did not improve. Hence, we decided to perform a left mastectomy under local anesthesia in the intensive care unit (ICU) to reduce the tumor volume. The majority of the tumor was necrotic, and the tissue was fragile. There was no obvious evidence of pectoralis major muscle involvement. Four days after admission, the acidosis did not improve and liver failure progression was observed. Hemodialysis was not expected to be effective; after consultation with her family, dialysis was discontinued, and the patient died 2 h later.

**Table 1:** Blood work during hospitalization showing elevated uric acid, acute renal failure, electrolyte abnormalities consistent with tumor lysis syndrome

	Reference range	Baseline prior to letrozole	Admission(hospital day 0)	Day 1	Day 2	Day 3
Sodium	135–145 mmol/L	135.1	127.8	132	151.3	139.7
Potassium	3.2–5.2 mmol/L	4.9	5	6.9	4.5	3.9
Blood urea nitrogen	8–20 mg/dL	30.7	77.2	88	84.7	26.8
Creatinine	0.4–0.8 mg/dL	0.94	2.08	2.64	2.57	0.97
Calcium	8.5–10.2 mg/dL	8.7	8.5	7.8	7	7.7
Phosphate	2.8–4.6 mg/dL	3.7	4	7.8	8.7	4.6
Uric acid	3.0–7.0 mg/dL	8.4	14.1	13.8	5.5	0.4
Lactate dehydrogenase	120–220 U/L	134	135	279	635	1904

Elevated uric acid, acute renal failure, and electrolyte abnormalities consistent with tumor lysis syndrome were observed

## Discussion

TLS is a potentially lethal oncological emergency in which massive tumor cell destruction causes severe electrolyte and metabolite abnormalities secondary to the release of intracellular components into the bloodstream, resulting in hyperuricemia, hyperkalemia, hyperphosphatemia, and secondary hypocalcemia. Hyperuricemia and hyperphosphatemia induce acute renal injury owing to uric acid precipitation and calcium phosphate deposition in the renal tubules. Hypocalcemia and hyperkalemia can also cause electrocardiographic abnormalities, arrhythmias, neuromuscular symptoms, and seizures. Following the introduction of the Cairo–Bishop definition, proposed in 2004, which provides TLS diagnostic criteria, TLS can now be diagnosed clinically, using laboratory values [5, 6]. The present patient met the Cairo–Bishop definitions of laboratory and grade II clinical TLS. After admission, the patient was treated with a high volume of rehydration fluid, glucose insulin therapy for hyperkalemia, and rasburicase for hyperuricemia. On the second day after admission, the patient developed progressive metabolic acidosis, and hemodialysis was initiated. On the third day after admission, the patient’s condition worsened. At this point, we considered the presence of the tumor to be related to the worsening condition; thus, we performed an emergency mastectomy under local anesthesia in the ICU. Unfortunately, the patient did not respond to these treatments and eventually died. At this point, we considered that the presence of the tumor was associated with worsening of the condition. Therefore, although not standard of care, we performed an emergency mastectomy under local anesthesia in the ICU. Unfortunately, the patient did not respond to these treatments and ultimately died.

There were two reasons why we decided to perform surgery. The first reason was that the patient’s general condition had deteriorated to the point where she developed consciousness disorders, and there was no other systemic treatment available. The second reason was that tumor removal might have been significant, considering the mechanism of tumor lysis syndrome. However, there have been no reports on the effectiveness of surgical resection of tumors in the treatment of tumor lysis syndrome, so it remains unclear whether this procedure was appropriate.

In the present case, although the patient had a solid tumor and was at a low risk for TLS, a prophylactic uric acid-lowering drug may have been indicated because of the large tumor volume and slightly elevated uric acid level (8.4 mg/dl prior to treatment). Therefore, control of elevated uric acid levels is important to prevent renal dysfunction. Furthermore, recognizing the high risk of TLS and assessing the risk factors prior to treatment are of utmost importance. In the present case, the high efficacy of letrozole was likely the trigger for TLS although the possibility of renal failure due to Mohs paste cannot be ruled out. In particular, clinical TLS reportedly increases mortality rates (83 vs. 24%; *p* < 0.001) [7]. The development of acute kidney injury associated with TLS is a strong predictor of mortality [8]. Regardless of cancer type, the mortality rate increases by 20–50% in cases of undiagnosed or delayed TLS diagnosis in solid tumors [9]. The best TLS management is prevention. Omori et al. [10] previously reported that prophylactic infusion and lowering uric acid levels prevented TLS in patients with breast cancer with high tumor volumes and hyperuricemia.

TLS has also been commonly reported in hematological malignancies but is becoming more frequently noted in solid tumors as treatments become more efficient [11]. Table 2 (modified from Watkinson and Hari Dass [3]) summarizes all reported TLS cases caused by breast cancer treatment. A total of 22 TLS cases associated with breast cancer have been reported, including three with hormone therapy only (one with tamoxifen and two with letrozole), three with hormone therapy plus a cyclin-dependent kinase 4/6 inhibitor or PIK3CA inhibitor, two with anti-HER2 therapy, nine with chemotherapy, two with radiation therapy, and three without therapy. Overall, chemotherapy, hormone therapy, molecular-targeted drug therapy, radiation therapy, and no therapy can all cause TLS.

**Table 2:** A Summary of previously published reports of TLS and hyperuricemia cases

Year of publication	Author	Age	Diagnosis	Current treatment	Risk factors specified	Outcome
2023	Furusawa M et al. [12]	65	Invasive ductal carcinoma ER + , PR + , HER2-, MIB-1 Index: 60%)	Palliative radiotherapy	Widespread metastases	Developed TLS after 11 h, and recovered 6 days later
2021	Handy C et al. [13]	66	Advanced, ER-, HER2- ductal tumor	PIK3CA + fulvestrant/alpelisib	Widespread metastases	Developed TLS on D12, and recovered 6 days later
2021	Watkinson GE et al. [3]	74	Occult breast cancer, ER + , HER2 −	Letrozole	Pleural seeding	Developed TLS on D3, and died 2 days later
2020	Carrier X et al. [14]	55	Advanced, ER-, HER2-, ductal tumor	Atezolizumab/nab-paclitaxel	Bone and liver and lung metastasis	Developed TLS on D6 Was discharged to home
2019	Aslam et al. [15]	58	Invasive poorly differentiated ductal carcinoma with widespread metastases. ER − , PR − , HER +	Gemcitabine	Widespread metastases	Developed TLS on D4. Was discharged to hospice
2019	Parsi et al. [16]	36	Grade-4 invasive ductal carcinoma. ER + , PR + , HER2 +	No treatment started before developing TLS	Widespread innumerable metastases	Spontaneous development of TLS after diagnosis of breast cancer but before starting treatment. Recovery from acute TLS with IV fluids and rasburicase
2019	Idrees et al. [17]	48	Infiltrating ER + , PR + , HER2 − , p53 − , Ki67 10% carcinoma with bony and liver metastases	No treatment had been given before TLS	Bony and liver metastases	Presentation with abdominal pain and oliguria. Found to be in spontaneous TLS from an as-yet undiagnosed breast cancer with widespread metastatic disease
2016	Bromberg et al. [18]	78	Advanced, ER + , HER2 − ductal tumor	Palbociclib/letrozole	Widespread metastases	Severe kidney injury resolved with intravenous fluids and allopurinol. Palbociclib dose reduced for ongoing treatment
2016	Bromberg et al. [18]	86	Advanced, ER + , HER2 − ductal tumor	Palbociclib/letrozole	Liver metastases	Developed hyperuricaemia was given allopurinol and encouraged to increase oral fluid intake. Uric acid level reduced and palbociclib restarted at lower dose
2016	Baudon et al. [19]	58	Invasive grade-III ductal carcinoma, ER − , PR − , HER2 − . Locally advanced and very widespread bony metastases	Trastuzumab and pertuzumab	High LDH at start of treatment and reduced eGFR (53). Widespread disease	Developed organ failure on D2 and died 48 h later from multiorgan dysfunction
2014	Vaidya and Acevedo [20]	52	Locally recurrent, invasive ductal cell carcinoma, ER + , PR + , HER2 −	Single dose paclitaxel	Liver metastases	Became encephalopathic on D7 and died during hemofiltration
2013	Taira et al. [21]	69	Invasive ductal carcinoma, triple-disease, T2N1M0, stage IIB	Trastuzumab	Liver metastases present	Developed a cardiac arrhythmia on D6 of trastuzumab, died from acute renal failure on D11
2005	Mott et al. [1]	44	Metastatic ER + , PR + and HER2 overexpressing carcinoma	Gemcitabine and cisplatin		Developed nausea and vomiting. Fluids and allopurinol then rasburicase given, full recovery of renal function made
2005	Mott et al. [1]	47	Stage I, ER + , PR − , HER2 − cancer diagnosed 4 years earlier	5-Fluorouracil, epirubicin, cyclophosphamide		Developed TLS 24 h into treatment. Fluids given with allopurinol and renal function gradually improved
2004	Kurt et al. [22]	42	Invasive ductal carcinoma, stage IIB	Capecitabine	Liver metastases present	11 h into capecitabine became confused, bradycardic, and oliguric. GCS 11 and died shortly after
2001	Zigrossi et al. [2]	61	Invasive ductal cell carcinoma, T2N0. ER + , PR +	Letrozole		Developed TLS on D2. Letrozole held, supportive management given was alive 20 months later
2000	Rostom et al. [23]	73	Male patient with widespread LN infiltration and bony disease	Hemibody irradiation	Widespread metastases	Developed renal failure 48 h after irradiation treatment, failed to respond to allopurinol and IV fluids, developed coma, and died 5 days following treatment
1997	Ustündağ et al. [24]	56	Tumor type not specified	Paclitaxel	Metastases, pre-existing elevated LDH	Became oliguric during first infusion and became confused. Started hemofiltration but died within 24 h from cardiac arrest
1995	Sklarin and Markham [25]	62	Infiltrating lobar carcinoma, with lung, liver, and bone metastases	No treatment before TLS diagnosis. Then recurred with	Bony and liver metastases	Already fulfilling criteria for TLS at time of initially presented with breast mass. Further episode of TLS following treatment with dibromodulcitol, doxorubicin, vincristine, tamoxifen, Halotestin, methotrexate, and leucovorin
1994	Drakos et al. [26]	31	Infiltrating ductal carcinoma, T2N1M0, ER +	Mitoxantrone	Liver metastases. Normal renal function	Developed biochemical and clinical TLS on D3. Died 1 month later from hepatic failure secondary to infiltrative disease
1987	Stark et al. [27]	53	Infiltrating ductal adenocarcinoma, ER + , PR −	5-Fluorouracil, docetaxel, cyclophosphamide	Extensive metastases, very elevated LDH, and raised urea	18 h posttreatment developed TLS, suffered cardiac arrest 48 h later
1986	Cech et al. [28]	94	Infiltrating ductal carcinoma. Hormone profile not performed	Tamoxifen	Extensive metastatic disease including widespread bony metastases	Renal function deteriorated on D7. Patient died 2 months later from congestive cardiac failure

The above cases were those available in the literature during publication of this report. The table details the publication author, patient age, any tumor type and stage specifics (where reported in the publication), treatment administered, risk factors specified, and outcome. Modified from Watkinson and Hari Dass [3]

*TLS* tumor lysis syndrome

## Conclusions

Herein, we describe the third reported TLS case in a patient with locally advanced breast cancer who developed the syndrome after receiving letrozole. Oncologists treating patients with breast cancer should be extremely cautious when treating patients with a high TLS risk, even without cytotoxic chemotherapy. As TLS can cause fatal outcomes, physicians should consider the risks and determine the appropriate prophylaxis before initiating treatment.

## Data Availability

Not applicable.

## Abbreviations

BUN: Blood urea nitrogen
Cr: Creatinine
TLS: Tumor lysis syndrome
HER2: Human epidermal growth factor receptor 2
ICU: Intensive care unit
